# A Longitudinal Study of Indoor Nitrogen Dioxide Levels and Respiratory Symptoms in Inner-City Children with Asthma

**DOI:** 10.1289/ehp.11349

**Published:** 2008-07-23

**Authors:** Nadia N. Hansel, Patrick N. Breysse, Meredith C. McCormack, Elizabeth C. Matsui, Jean Curtin-Brosnan, D’Ann L. Williams, Jennifer L. Moore, Jennifer L. Cuhran, Gregory B. Diette

**Affiliations:** 1 Department of Medicine, School of Medicine, Johns Hopkins University, Baltimore, Maryland, USA; 2 Department of Environmental Health Sciences, Bloomberg School of Public Health, Johns Hopkins University, Baltimore, Maryland, USA; 3 Department of Pediatrics, School of Medicine, Johns Hopkins University, Baltimore, Maryland, USA

**Keywords:** asthma, indoor pollutants, inner city, nitrogen dioxide, preschool

## Abstract

**Background:**

The effect of indoor nitrogen dioxide concentrations on asthma morbidity among inner-city preschool children is uncertain.

**Objectives:**

Our goal was to estimate the effect of indoor NO_2_ concentrations on asthma morbidity in an inner-city population while adjusting for other indoor pollutants.

**Methods:**

We recruited 150 children (2–6 years of age) with physician-diagnosed asthma from inner-city Baltimore, Maryland. Indoor air was monitored over a 72-hr period in the children’s bedrooms at baseline and 3 and 6 months. At each visit, the child’s caregiver completed a questionnaire assessing asthma symptoms over the previous 2 weeks and recent health care utilization.

**Results:**

Children were 58% male, 91% African American, and 42% from households with annual income < $25,000; 63% had persistent asthma symptoms. The mean (± SD) in-home NO_2_ concentration was 30.0 ± 33.7 (range, 2.9–394.0) ppb. The presence of a gas stove and the use of a space heater or oven/stove for heat were independently associated with higher NO_2_ concentrations. Each 20-ppb increase in NO_2_ exposure was associated significantly with an increase in the number of days with limited speech [incidence rate ratio (IRR) = 1.15; 95% confidence interval (CI), 1.05–1.25], cough (IRR = 1.10; 95% CI, 1.02–1.18), and nocturnal symptoms (IRR = 1.09; 95% CI, 1.02–1.16), after adjustment for potential confounders. NO_2_ concentrations were not associated with increased health care utilization.

**Conclusions:**

Higher indoor NO_2_ concentrations were associated with increased asthma symptoms in preschool inner-city children. Interventions aimed at lowering NO_2_ concentrations in inner-city homes may reduce asthma morbidity in this vulnerable population.

Asthma is the most common chronic disease of childhood, affecting 6.5 million (8.9%) children in the United States and disproportionately causing morbidity in inner-city, minority children (Bloom et al. 2006). Many factors have been examined, and although no single explanation exists to explain such racial disparities, evidence supports a multitude of risk factors including differing exposure to environmental pollutants and differences in susceptibility (Barnes et al. 2007; Weiss et al. 1992). Several pollutants have been shown to worsen asthma including particulate matter (PM), ozone, and nitrogen dioxide (Institute of Medicine Committee on the Assessment of Asthma and Indoor Air 2000; Koren 1995). NO_2_ may be a particular problem in the inner city, where gas stoves are common and proper venting of stoves may be rare. In fact, our group has previously demonstrated high indoor NO_2_ concentrations in inner-city homes (Breysse et al. 2005; Diette et al. 2007). Furthermore, because preschool children spend much of their time in the home (Klepeis et al. 2001; McCormack et al. 2007a), they may be especially at risk to the adverse effects of indoor NO_2_ exposure.

Although some studies have shown that NO_2_ can affect asthma in children (Belanger et al. 2006; Garrett et al. 1998; Hasselblad et al. 1992; Kattan et al. 2007; Nitschke et al. 2006; Shima and Adachi 2000; Smith et al. 2000), very few have focused on African-American, inner-city children or preschool children. It is important to replicate findings from previous studies done in other populations because some results have been inconsistent (Florey et al. 1979; Hoek et al. 1984; Samet et al. 1993; Sunyer et al. 2004), and future guidelines and public policies depend on a robust evidence base. The purpose of this study was to investigate the independent longitudinal effect of indoor NO_2_ concentrations on asthma morbidity, accounting for copollutants. To study this question, we conducted a prospective cohort study of predominantly African-American preschool children with asthma living in inner-city Baltimore, Maryland.

## Methods

Participants were recruited for the Baltimore Indoor Environment Study of Asthma in Kids as previously described (Hansel et al. 2006; McCormack et al. 2007a; Sharma et al. 2007). All subjects were residents of inner-city Baltimore, defined by nine contiguous ZIP codes and encompassing a relatively small area (approximately 4 mi^2^). Potential participants were identified from a random sample of children with a health care encounter for asthma in the previous 12 months at Johns Hopkins Community Physicians or Bayview Pediatrics. Eligibility criteria were as follows: *a*) age between 2 and 6 years, *b*) physician-diagnosed asthma, *c*) at least one health care encounter for asthma within the preceding 12 months (according to the *International Classification of Diseases, 9th Revision*, code 493.xx) (World Health Organization 2000), and *d*) symptoms or use of asthma medications within the preceding 6 months. All participants were told the general purpose of the study, including the study of the effects of the home environment on asthma health, but were not aware of the specific study hypotheses addressed here. The Johns Hopkins Medical Institutional Review Board approved the study protocol.

Participants underwent home visits at baseline and 3 and 6 months to complete health and environmental surveys, home inspection, and environmental sample collection.

### Patient characteristics

The health questionnaire included questions assessing demographics, comorbidities, and medication use and modified questions from the International Study of Asthma and Allergies in Childhood (Asher et al. 1995) and the Children’s Health Survey for Asthma (Asmussen et al. 1999) to evaluate indicators of poor asthma control. Caregivers were asked about their child’s symptoms in the previous 2 weeks regarding *a*) any daytime wheezing, coughing, or tightness in the chest; *b*) need to slow down or stop activities at home or playing because of asthma, wheezing, or tightness in the chest or cough; *c*) wheezing that was so bad that he or she could speak only one or two words at a time between breaths; *d*) wheezing, coughing, or tightness in the chest when running or going up stairs; *e*) coughing that wasn’t from a cold; and *f)* nocturnal awakenings because of cough, wheeze, shortness of breath, or tightness in chest. Each symptom was quantified as the number of days that the symptom was present (0–14 days). Questions also ascertained information about acute health care use for asthma in the preceding 3 months (emergency department visits, unscheduled doctor visits, and hospitalizations) and rescue medication use in the previous 2 weeks (short-acting beta agonist). Child’s asthma severity was categorized as recommended by the National Asthma Education and Prevention Program guidelines (EPR-2) (National Heart, Lung, and Blood Institute 1997). Allergic sensitization status was assessed by skin prick test to 14 common aero-allergens. A child was considered atopic if he or she had at least one positive (wheal diameter ≥2 mm) skin test.

### Environmental assessment

We used an interviewer-administered environmental questionnaire to assess housing characteristics and potential sources of indoor NO_2_ concentrations. A time–activity diary was administered during the 72-hr monitoring period to track household activities that may be correlated with indoor NO_2_ concentrations, including number of windows open for > 10 min/day, vacuuming, sweeping, and stove/oven, space heater, candle/incense, air purifier, and cigarette use. A home inspection was conducted to assess distance from curb to front door of the home and type of street in front of house (side street, arterial, or parking lot).

Indoor air sampling for NO_2_ and PM_2.5_ (PM with an aerodynamic diameter ≤2.5 μm) was conducted continuously over a 72-hr period, as previously described (Diette et al. 2007; McCormack et al. 2007a). The monitors were placed in the sleeping room of the child, which was ascertained by the technician before placement of the monitors. All sampling heads and passive badges were attached to the outside of a sampling frame that was placed in a convenient location in the child’s bedroom. In most cases, the sampling frame was placed on the dresser or a nightstand. In some cases, when there was no available elevated surface, the sample frame was place on a portable stand constructed out of polyvinyl chloride pipe. NO_2_ was measured using a passive sampler (Ogawa badge) loaded with filters coated with triethanolamine (TEA) (Palmes et al. 1976). Samplers and coated filters were purchased from Ogawa, Inc. (Pompano Beach, FL). In the presence of a color reagent, NO_2_ and TEA form a highly colored azo dye that is measured spectrophotometrically at 540 nm. The median limit of detection calculated from the analysis of field blanks was 6.8 ppb. PM_2.5_ samples were collected using 4 L/min MSP impactors (MSP Corp., St. Paul, MN) loaded with 37-mm, 2.0-μm pore size, PALL Teflo PTFE membrane filters with polypropylene support rings (Pall Corp., Ann Arbor, MI).

Daily ambient NO_2_ levels during the study period were obtained from the U.S. Environmental Protection Agency Air Quality System database (U.S. Environmental Protection Agency 2007) All homes were within 4.8 miles of the central monitoring site.

### Statistical analysis

We used descriptive statistics to characterize the patient sample and the pollutant levels. We compared summary statistics using Spearman correlations, chi-square tests for proportions, and *t*-tests for continuous data. All variables had < 10% missing data; therefore, missing values were imputed with the median or mode for continuous or categorical variables, respectively.

Adjusting for season of sampling, we used linear regression models to assess whether household characteristics and daily activities during the monitoring period predicted baseline indoor NO_2_ concentrations. Variables with *p* ≤ 0.05 were included in a multivariate model to assess the independent effect of each predictor. To evaluate the effect of NO_2_ on asthma health, we analyzed NO_2_ as a continuous variable using negative binomial or logistic regression models and generalized estimating equations (Diggle et al. 2002) to estimate incidence rate ratios (IRRs) and odds ratios (ORs), respectively, and to take into account the intercorrelation arising from repeated measures over time. At each time point (baseline, 3 months, and 6 months), the mean NO_2_ concentration over the 3-day monitoring period and symptom frequency reported over the previous 2-week period were used as exposure and outcome variables, respectively. We used multivariate models to adjust for potential confounders, including age, sex, race, caregiver education level, season of sampling, PM_2.5_, secondhand smoke (SHS) exposure [defined as caregiver report of presence of a smoker in the home (yes vs. no)], distance from curb, and type of street in front of house. We conducted additional analyses including mean ambient NO_2_ as a covariate to ensure that the effects of indoor NO_2_ were independent of ambient NO_2_ levels. Stratified analyses were performed separately on subjects with atopy and those using daily inhaled corticosteroids (ICS). All analyses were performed with StataSE statistical software, version 8.0 (StataCorp, College Station, TX). Statistical significance was defined as *p* < 0.05.

## Results

### Participant characteristics

Participants were preschool children with asthma, most of whom were atopic (69%) and had persistent asthma symptoms at baseline (Table 1). Approximately 80% of the primary caregivers had no more than a high school education. Children were predominantly African American, and many children (42%) lived in households with incomes < $25,000, representing an urban population of low socioeconomic status. Eighty-three percent of participant homes had gas stoves, and 72% were heated by natural gas fuel. As ascertained by the daily time–activity diary, when home the children spent most of their time in the room where the monitoring occurred. Specifically, children in this study, on average, spent 13 hr/day in their own home, and 7 hr/day in the bedroom where monitoring occurred.

### Indoor NO_2_ concentrations

**Figure 1:**
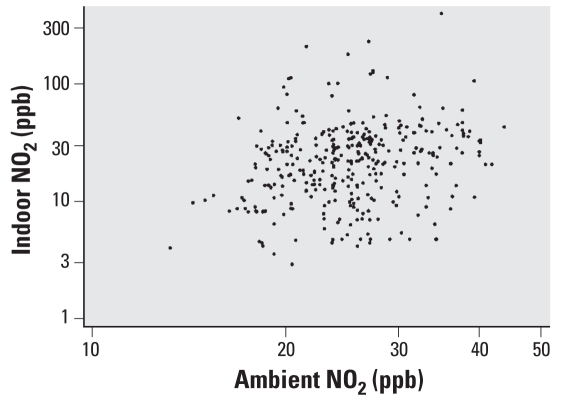
Correlation between indoor and ambient NO_2_ concentrations. Mean ambient and indoor NO_2_ concentrations during the monitoring period are represented in parts per billion on the *x*- and *y*-axes, respectively. There was minimal correlation between indoor and outdoor concentrations (Spearman’s *r*^2^ = 0.056, *p* < 0.01).

Most of the homes were row homes (homes that share adjacent walls; 79%) and close to the street (within 25 feet; 71%). Sixty-two percent of homes were in front of a side street, 27% in front of an arterial street, and 11% in front of a parking lot. The overall mean (± SD) indoor NO_2_ concentration was 30.0 ± 33.7 ppb (range, 2.9–394.0 ppb), and mean PM_2.5_ concentration was 40.3 ± 35.4 μg/m^3^ (range, 0.1–216.1 μg/m^3^). There was no statistical difference in mean pollutant concentrations between baseline, visit 2, and visit 3. NO_2_ concentrations were significantly lower in summer (15.9 ± 14.0 ppb) than in any other season (fall: 30.8 ± 27.5 ppb; winter: 41.4 ± 52.1 ppb; spring: 30.7 ± 23.5 ppb; *p* < 0.001). The mean ambient NO_2_ concentration during the study period was 25.7 ppb. There was minimal correlation (*r*^2^ = 0.056, *p* < 0.01) between ambient and indoor NO_2_ concentrations (Figure 1).

NO_2_ concentrations were higher in homes with a gas stove (mean, 33.1 ppb) compared with those without a gas stove (mean, 16.8 ppb). Similarly, the mean indoor NO_2_ concentrations were 7.2 ppb higher in homes with a gas heater compared with those without a gas heater, and the presence of a gas heater had a greater effect on indoor NO_2_ concentrations during the winter months (β = 17.8; SE 9.7). In addition, after adjusting for season, using a space heater or the stove or oven for heat during the monitoring period was associated with higher NO_2_ concentrations. The independent effect of the presence of a gas stove and using a space heater or the stove or oven for heat on indoor NO_2_ concentrations remained essentially unchanged after adjustment for the presence of the other predictors. Other housing characteristics and daily activities were not associated with indoor NO_2_ levels (Table 2).

### Association of NO_2_ levels with asthma outcomes

Higher NO_2_ concentrations were associated with statistically significant increases in respiratory symptoms (Table 3). After adjusting for potential confounders, increasing NO_2_ concentrations remained significantly associated with increasing frequency of limited speech due to wheezing, coughing without a cold, and nocturnal awakenings due to cough, wheeze, and shortness of breath or chest tightness during the daytime and while running. There was no significant relationship between NO_2_ concentration and rescue medication use or health care utilization [rescue medication use OR = 1.00 (95% confidence interval [CI], 0.93–1.07); unscheduled doctor’s visit OR = 0.99 (95% CI, 0.92–1.35); asthma-related hospital visit OR = 1.13 (95% CI, 0.69–1.09); emergency department visit for asthma OR = 0.88 (95% CI, 0.83–1.17)].

In general, the presence of atopy did not modify the effect of NO_2_ exposure on asthma symptoms, except that individuals with atopy were more likely to experience nocturnal symptoms with increasing NO_2_ concentration (IRR = 1.13 per 20-ppb increase in NO_2_) compared with nonatopic individuals (IRR = 1.03). In addition, daily use of ICS did not modify the association of NO_2_ concentrations and asthma symptoms, and mean ambient NO_2_ concentrations were not significantly associated with any respiratory symptoms (data not shown).

## Discussion

Our study shows that exposure to increasing concentrations of indoor NO_2_ is associated with increased respiratory symptoms among a group of predominantly African-American, inner-city, preschool children with asthma. Understanding the effect of indoor air quality on asthma morbidity in inner-city preschool children is necessary because preschool children spend most of their time indoors (Klepeis et al. 2001), and inner-city minority children suffer disproportionately from asthma and are exposed to high levels of indoor pollutants (Bloom et al. 2006; Breysse et al. 2005). Specifically, many inner-city households use gas stoves, an important source of indoor NO_2_ concentrations, and many of these stoves are unvented (Breysse et al. 2005; Diette et al. 2007). Furthermore, almost 14% of the homes in this study used gas stoves for heat. Because the use of a stove as a source of heat is seen almost exclusively in the context of profound poverty, this study also highlights the complex interaction of poverty with environmental exposures in an inner-city minority population.

Current evidence has not yet convincingly demonstrated that high indoor NO_2_ concentrations contribute to the risk of developing asthma, because NO_2_ concentrations are similar in homes of children with and without asthma (Diette et al. 2007; Hoek et al. 1984; Institute of Medicine Committee on the Assessment of Asthma and Indoor Air 2000). Studies done in subjects with asthma have suggested that higher indoor NO_2_ concentrations lead to increased asthma symptoms; however, results have not been consistent across subpopulations (Belanger et al. 2006; Garrett et al. 1998; Hasselblad et al. 1992; Kattan et al. 2007; Nitschke et al. 2006; Shima and Adachi 2000; Smith et al. 2000). Young children and those with lower socioeconomic status (SES) may be at particular risk. For example, Smith et al. (2000) identified an association between NO_2_ concentrations and increased risk of asthma symptoms in individuals < 14 years of age, but not in older individuals, except for a marginal increased risk of cough in subjects 35–49 years of age. In a cross-sectional analysis, Belanger et al. (2006) found that indoor NO_2_ exposure was associated with increased incidence and frequency of chest tightness and wheezing, but only in individuals living in multifamily housing units, which was an indicator of lower SES. Our longitudinal study with repeated measures of NO_2_ concentrations and respiratory symptoms improves on our ability to directly model individual response to changing NO_2_ concentrations accounting for within-person correlations of asthma severity. Our results show a consistent link between increased NO_2_ concentrations and increased respiratory symptoms in preschool children with asthma. We further show that inner-city, predominantly minority children are exposed to high levels of indoor NO_2_ concentrations. The burden of asthma attributable to differences in domestic NO_2_ concentrations is substantial. For example, a child would experience 10% more days of cough symptoms or 15% more days with limited speech due to wheeze with each 20-ppb increase in NO_2_ exposure. A child experiencing limited speech once a week would experience an additional 13 days of limited speech per year if living in a home where NO_2_ levels increased by 33.6 ppb (1 SD). Importantly, NO_2_ was consistently associated with coughing, nocturnal symptoms, and limited speech, even after adjusting for potential confounders and other pollutants.

The indoor NO_2_ concentrations observed in this study are high compared with levels found in some other studies, with mean indoor NO_2_ concentrations in most studies ranging between 6 and 30 ppb (Nitschke et al. 1999). However, the levels we found are consistent with levels measured in other studies that have focused on inner-city homes (Breysse et al. 2005; Kattan et al. 2007; Zota et al. 2005). Because the presence of a gas stove or gas heater and the use of a space heater or gas or oven for heat were associated with higher NO_2_ concentrations, and ambient NO_2_ concentrations were only minimally correlated with indoor levels, it appears that changes to home heating and cooking devices may be a feasible means to reduce the burden of asthma. To our knowledge, there has been only one previous trial specifically targeting NO_2_ (Pilotto et al. 2004). In that study, conducted in schools, unflued gas heaters were replaced with either electric heaters or flued gas heaters. The intervention led to reduced NO_2_ concentrations and respiratory symptoms in children with asthma (Pilotto et al. 2004). We believe that clinical trials are still needed to assess the effectiveness of reducing NO_2_ concentrations in inner-city homes on improving asthma morbidity.

The link between indoor NO_2_ concentrations and asthma symptoms appears to be robust, because the associations were not significantly affected by the potential confounders studied. Indeed, our study was strengthened by its ability to adjust for other relevant copollutants. Although PM_2.5_ was associated with increased asthma symptoms (McCormack et al. 2007b) (data not shown), adjusting for other copollutants did not meaningfully alter the association between indoor NO_2_ concentrations and asthma symptoms. However, because ambient NO_2_ concentrations can vary within a community based on traffic-related exposure (Clougherty et al. 2008; Gauderman et al. 2005; Wheeler et al. 2007), residual confounding of traffic-related exposure may lead to misclassification of individual ambient NO_2_ exposure. Additionally, stratified analyses showed that the associations between NO_2_ exposure and asthma symptoms were not significantly different in atopic subjects and those with daily ICS use. These findings are in contrast to the results of the National Cooperative Inner-City Asthma Study in which an association of higher levels of indoor NO_2_ with increased asthma symptoms was found only in nonatopic children (Kattan et al. 2007). Indoor NO_2_ concentrations were not associated with health care utilization. Thus, it is possible that indoor NO_2_ exposure was related to increased respiratory symptoms but not sufficient to precipitate severe asthma attacks requiring unscheduled doctor visits, emergency department visits, or hospitalizations. In addition, the link between indoor NO_2_ concentrations and respiratory symptoms is not corroborated with objective data on pulmonary function, given the difficulty in obtaining reliable measures of lung function in this young age group. It is also possible that our study under- or overestimated the true effect of indoor NO_2_ concentrations on asthma symptoms, because we did not include personal monitoring of exposure. However, we are reassured that children in the present study spent more than half their time in their own home, most of which was in the bedroom. Furthermore, studies have shown that indoor NO_2_ concentrations remain relatively stable in a given home over a month-long period (Brunekreef et al. 1987; Spengler et al. 1996), suggesting that short-term monitoring is a reasonable reflection of recent exposure levels.

In summary, in this study we found a link between higher NO_2_ concentrations and increased respiratory symptoms in preschool, inner-city, primarily African-American children with asthma. Furthermore, individuals in the inner city appear to be at particularly high risk for the adverse effects of indoor NO_2_ concentrations, given their high indoor exposure levels. The presence of a gas stove, as well as use of stove/oven and space heater for heat, was independently associated with higher indoor NO_2_ levels, which suggests that there are modifiable sources of exposure. Our study has major implications for health care providers and asthma patients. The next step should be to conduct research studies that show whether changing from natural gas to other fuel sources (e.g., electricity) can reduce the asthma burden. In the meantime, we would recommend that families who have children with asthma, if afforded the opportunity to choose housing, choose homes without gas stoves and heaters. Those who already have these appliances should be cautioned at least about the need for proper venting of the exhaust gases. Interventions aimed at lowering NO_2_ concentrations in inner-city homes may reduce asthma morbidity in this vulnerable population.

## Figures and Tables

**Table 1:** Child and caregiver characteristics (*n* = 150).

Characteristic	Percent
Child characteristics	
Age [mean (range)]	4.4 (2–6)
Male sex	58.0
Race	
Black	91.2
White	4.7
Other	4.1
Asthma severity	
Mild intermittent	37
Mild persistent	17
Moderate persistent	21
Severe persistent	25
Atopic	69
Asthma medication use (last 2 weeks)	
Albuterol	53
ICS	34
Other (cromolyn, leukotriene modifier, theophylline, oral corticosteroids)	19
Caregiver characteristics	
Primary caregiver	
Birth mother	87.1
Grandmother	4.8
Birth father	2.7
Other	5.4
Education	
Not high school graduate	38.5
High school graduate	42.6
At least some college	19.1
Household income (annual)	
< $25,000	41.6
$25,000–$50,000	10.8
> $50,000	2.0
Not reported	20.8

**Table 2:** Predictors of indoor NO_2_ concentrations (adjusted for season).

					Multivariate models^b^		
Characteristic	Percent reporting characteristic or activity^a^	β	95% CI	*p*-Value	β	95% CI	*p*-Value
Housing characteristics							
Gas stove	83	15.0	6.2 to 23.8	< 0.001	15.7	6.9 to 24.6	0.001
Gas heater	72	7.2	−0.1 to 14.6	0.05	4.4	−2.8 to 11.6	0.23
Daily activities over the monitoring period							
Space heater use	5	16.40	1.19 to 31.61	0.04	14.4	0.8 to 28.8	0.05
Stove/oven for heat	12	12.49	2.44 to 22.53	0.02	12.4	2.6 to 22.2	0.01
Sweeping (per sweeping event)	85	1.00	−0.12 to 2.11	0.08	[Table-fn tfn1-ehp-116-1428]—	[Table-fn tfn1-ehp-116-1428]—	[Table-fn tfn1-ehp-116-1428]—
Cigarettes (per cigarette)	56	0.04	−1.0 to 0.17	0.59	[Table-fn tfn1-ehp-116-1428]—	[Table-fn tfn1-ehp-116-1428]—	[Table-fn tfn1-ehp-116-1428]—
Open windows (per open window)	85	−0.38	−1.09 to 0.33	0.29	[Table-fn tfn1-ehp-116-1428]—	[Table-fn tfn1-ehp-116-1428]—	[Table-fn tfn1-ehp-116-1428]—
Candles/incense	32	−2.37	−9.66 to 4.93	0.52	[Table-fn tfn1-ehp-116-1428]—	[Table-fn tfn1-ehp-116-1428]—	[Table-fn tfn1-ehp-116-1428]—
Air purifier use	1	−9.17	−49.38 to 31.03	0.65	[Table-fn tfn1-ehp-116-1428]—	[Table-fn tfn1-ehp-116-1428]—	[Table-fn tfn1-ehp-116-1428]—

—, no data are available for multivariate models for these variables because only variables with *p* < 0.05 on bivariate analyses were included in the multivariate model.

a All characteristics or activities are reported as present (yes vs. no) during the monitoring period, except for the number of cigarettes smoked, number of sweeping events, and number of open windows (for > 10 min) during the monitoring period, which were analyzed as continuous variables.

b Multivariate models are adjusted for presence of gas stove, presence of gas heater, use of space heater, use of oven/stove for heat and season.

**Table 3:** Risk of asthma symptoms per 20-ppb increase in NO_2_ exposure.

	Unadjusted		Adjusted^a^		Adjusted^b^	
Symptom	IRR	95% CI	IRR	95% CI	IRR	95% CI
Daytime wheezing, coughing, or chest tightness	1.05	0.99–1.12	1.03	0.96–1.11	1.04	0.97–1.12
Slowing activity due to asthma, wheeze, chest tightness, or cough	1.07	1.00–1.14*	1.06	0.99–1.14	1.08	0.94–1.15
Limited speech due to wheeze	1.12	1.04–1.21**	1.15	1.05–1.25**	1.17	1.08–1.27**
Wheeze, cough, or chest tightness while running	1.08	1.01–1.15*	1.07	0.99–1.14	1.09	1.01–1.17*
Coughing without a cold	1.13	1.06–1.20#	1.10	1.02–1.18**	1.15	1.07–1.23#
Nocturnal awakenings due to cough, wheeze, shortness of breath, or chest tightness	1.11	1.04–1.18#	1.09	1.02–1.16*	1.12	1.04–1.19**

a Models are adjusted for PM_2.5_; SHS; season of sampling; age, sex, and race of the child; and mother’s education level.

b Models are adjusted for PM_2.5_; SHS; distance from the curb, type of street in front of home, season of sampling; age, sex, and race of the child; and mother’s education level.

* *p* < 0.05.

** *p* < 0.01.

# *p* < 0.001.
